# Do Primitive Neuroectodermal Tumors of the Kidney Have a Predilection for Inferior Vena Cava Involvement? A Case Series and Review of the Literature

**DOI:** 10.15586/jkcvhl.2020.153

**Published:** 2020-10-12

**Authors:** Sovan Hota, Sidhartha Kalra, Lalgudi Narayanan Dorairajan, Ramanitharan Manikandan, Sreerag Kodakkattil Sreenivasan

**Affiliations:** Department of Urology and Renal Transplantation, JIPMER, Puducherry, India

**Keywords:** immunohistochemistry, IVC thrombus, multimodality treatment, PNET, renal pelvis PNET, renal PNET

## Abstract

The primitive neuroectodermal tumor (PNET) of the kidney is an extremely rare neoplasm, the diagnosis of which mainly depends upon histopathology, immunohistochemistry (IHC), and cytogenetics. A handful of cases reported in the literature mention about aggressive features of this neoplasm. The purpose of our study was to review our experience in not only the diagnosis and management of the patients with renal PNET but also to highlight its propensity to involve inferior vena cava (IVC) and also present a rare occurrence of Ewing’s sarcoma (ES)/PNET of the renal pelvis.

The clinical, operative, and histopathology records of four patients of renal PNET treated between January 2017 and December 2019 were reviewed and data analyzed concerning the available literature. Out of the four patients treated, two had level III and IV IVC thrombus, and one had dense desmoplastic adhesions with the IVC wall. One of the cases had a rare presentation of ES/PNET of the renal pelvis. All patients were managed surgically, while only one patient received adjuvant chemotherapy and following up with remission for the last 2 years and 4 months. On IHC, cluster of differentiation-99 (CD-99) was positive in all patients, and three were positive for Friend leukemia integration-1. PNET of the kidney is primarily an immunohistopathological diagnosis. This neoplasm has an increased propensity for the local invasion of surrounding structures. A multimodality approach with surgery, chemotherapy, and radiotherapy could offer better outcomes, although the prognosis of these tumors remains poor.

## Introduction

The primitive neuroectodermal tumor (PNET) includes a group of small round cell tumors (RCT) of universal location and apparent neuroectodermal origin, presenting as a disease of bone and soft tissue (1). In a rare situation, PNET has been found in the genitourinary system such as kidney (2), bladder (3), prostate (4), testis, epididymis, ovary, and uterus (5). Very rarely, they can occur as a primary renal tumor (6). Renal PNET exhibits highly aggressive biological behavior with poor prognosis, and until now <100 cases have been reported (7). Generally, renal PNET affects young adults at a median age of 28 years and has a male predominance of 3:1 (8). Histopathology and immunohistochemistry (IHC) showing positivity for cluster of differentiation-99 (CD-99) and Friend leukemia integration-1 (FLI-1), supported by cytogenetic studies, play a significant role in the diagnosis of PNET (9).

We herein report our experience of clinical presentation, imaging, and management of four cases of renal PNET involving inferior vena cava (IVC) and one out of these presenting as a renal pelvis tumor.

## Case series

From January 2017 to December 2019, four cases of renal PNET were managed in the department of urology and renal transplantation at our tertiary care institute. Consent from study participants and approval of the institutional ethics committee were taken to review the case records. The summary of clinical presentation, and treatment and follow-up are described in Table 1.

**Table 1:** Summary of clinical presentation, and treatment and follow-up of four cases of renal PNET.

Parameters	Case 1	Case 2	Case 3	Case 4
Age/sex	19/Male	30/Female	62/Female	56/Female
Presentation	M, P	P, M, W, H	W	P, F
Performance scale (ECOG)	1	2	2	2
Laterality	Right	Left	Right	Right
Extent	L	LA	LA	L
Provisional diagnosis	RCC	Renal oncocytoma+ ? coexistent RCC	RCC	TCC
Surgery	RN+LND	RN+LND+IVCT	RN+LND+IVCT	RN+LND+IVC cuff excision and primary repair
Complication Clavien–Dindo classification grades	I	V (perioperative mortality)	II (blood transfusion)	IIIa (wound dehiscence)
Stage	pT2bN0Mx Stage 2	pT4aN0Mx Stage 4	pT4aN0MX Stage 4	pT1N0Mx Stage 1
IHC	CD-99, FLI-1 positive CD-56, WT-1 negative	CD-99 positive FLI-1, WT-1, CK-1, Des-, BCL-2 negative	CD-99, FLI-1 positive CK-7, CK-20, p63, SNP-, CG-, NSE negative	CD-99, FLI-1 positive CD-56, WT-1 negative
Adjuvant therapy	Chemotherapy VAC/IE -17 cycles, G-CSF	No	No	No
Survival in months	2 years and 4 months with remission	Perioperative mortality	8 months	Lost to follow-up

PNET, primitive neuroectodermal tumor; ECOG, Eastern Cooperative Oncology Group; IHC, immunohistochemistry; G-CSF, granulocyte-colony stimulating factor; LA, locally advanced; RN, radical nephrectomy; VAC/IE, vincristine, adriamycin, cyclophosphamide/ifosfamide, etoposide; CG, chromogranin; CK, cytokeratin; Des, Desmin; NSE, neuron-specific enolase; SNP, synaptophysin; WT-1, Wilms tumor.

In our study, the median age was 43 years (age range 19–62 years). The most common complaint was abdominal pain in three (75%) cases, abdominal mass in two (50%) cases, weight loss in two (50%) cases, hematuria in 1 (25%) case, and fever in 1 (25%) case. None of the patients had evidence of metastasis.

**Figure 1:**
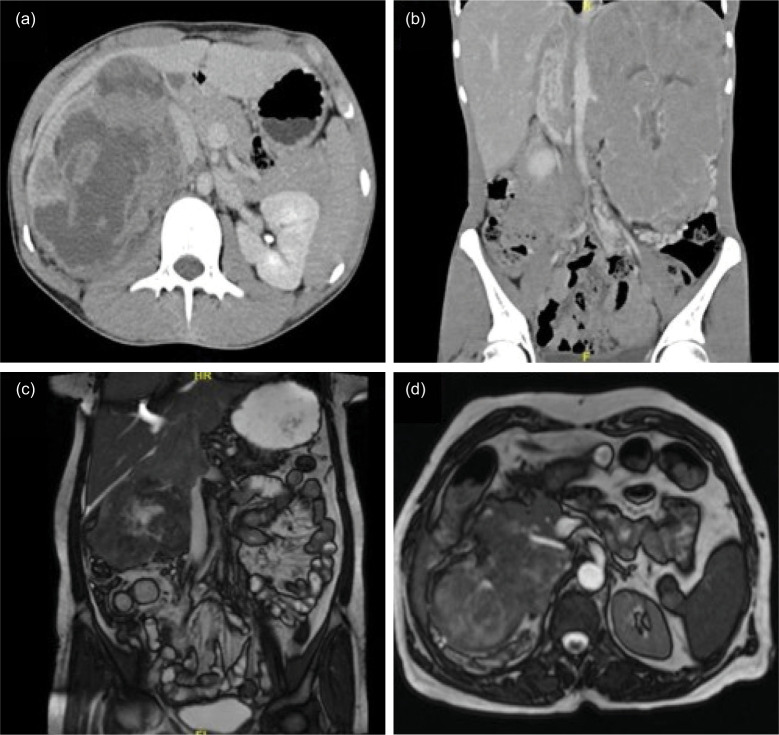
(a) CECT abdomen and pelvis transverse section (Case 1) showing right renal mass, compressing medially the right renal vein, ureter, and IVC with sectoral contact of about 180° with no apparent tumor thrombosis. (b) CECT thorax and coronal abdomen section (Case 2) revealed that left renal mass lesion with enhancing tumor thrombus extending up to the supradiaphragmatic IVC. (c) Plain MRI abdomen coronal section (Case 3) showing well-defined lobulated mass lesion with IVC thrombosis extending into the suprarenal IVC, below the diaphragm. (d) MR urography transverse section (Case 4) showing a well-defined mass of size 8 × 6 cm involving the renal pelvis, compressing IVC anteriorly. IVC, inferior vena cava; CECT, contrast-enhanced computed tomography.

Three of the cases had nonspecific imaging findings and were thought to be Renal cell carcinoma (RCC) provisionally (Figures 1a, b, and c). In contrast, another patient was diagnosed with an upper tract transitional cell carcinoma (TCC). Magnetic resonance (MR) urography of this case (Figure 1d) showed a heterogenous well-defined mass lesion of 8 × 6-cm size predominantly iso-intense in T2 and iso to hypo-intense in T1 with restricted diffusion, involving renal pelvis of the right kidney and anteriorly compressing IVC with focal loss of the fat plane. Gross hydronephrosis was noted with thinning of parenchyma along with deranged renal parameters. All cases underwent radical nephrectomy and lymph node dissection. The median size of the tumor was 14 cm (range 12–25.5 cm). Two patients underwent IVC thrombectomy. One patient, diagnosed with intrapericardial IVC thrombus, required cardiopulmonary bypass but had significant intraoperative blood loss because of extensive neovascularization. She developed disseminated intravascular coagulation (DIC) postoperatively and succumbed to the disease.

**Figure 2:**
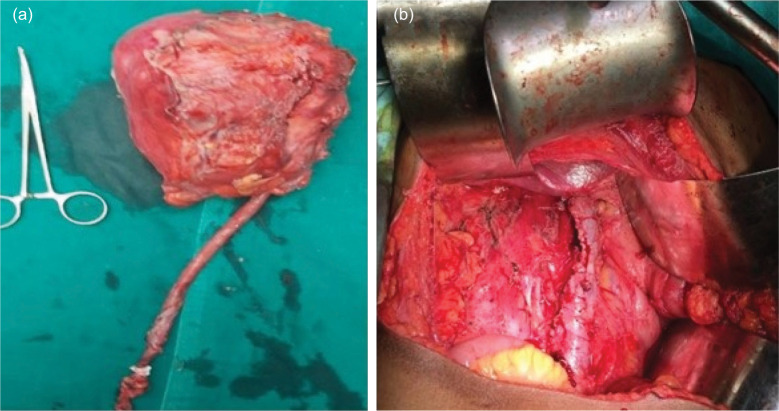
Intra-operative pictures of Case 4. (a) Showing tumor specimen of size 12.5 × 8 × 4 cm involving renal pelvis with grossly hydronephrotic right kidney. (b) Showing post-nephroureterectomy right renal bed with repaired IVC after excising involved cuff. IVC, inferior vena cava.

Intraoperatively, in the case of renal pelvis tumor, dense desmoplastic adhesions with IVC were found along the entire length of the mass (Figures 2a and b). She underwent radical nephroureterectomy, lymph node dissection, and IVC cuff excision with primary repair.

**Figure 3:**
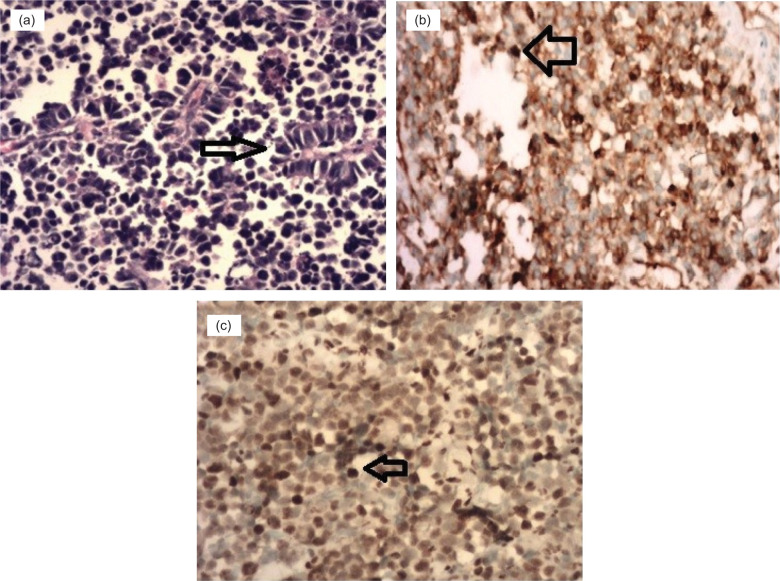
Histopathology and immunohistochemistry of case 4. (a) Showing (Hematoxylin–Eosin staining) small uniform round cells with a hyperchromatic nucleus and mild to moderate pale staining cytoplasm and pseudo-rosettes (20×). (b) Small round cells CD-99 IHC positive (20×). (c) Small round cells, FLI-1 positive (20×).

Histopathology and IHC study was done for all cases. It revealed tumor cells arranged in sheets. These comprise small, round-to-oval cells with a hyperchromatic nucleus and mild-to-moderate pale staining cytoplasm. At places, the tumor cells were arranged in pseudo rosettes (Figure 3a). On IHC, we have consistently found CD-99 in all cases (Figure 3b), FLI-1 in three (75%) cases (Figure 3c), and vimentin in one (25%) case. Provisionally diagnosed case of upper tract TCC was confirmed to be ES/PNET of renal pelvis origin along with the presence of infiltration of the ureter (Figures 3a, b, and c).

After discussion of institutional tumor board, one patient agreed to receive adjuvant chemotherapy with Vincristine, Adriamycin, Cyclophosphamide, Etoposide, and Ifosfamide (VAC/IE) of total 17 cycles as per Ewing’s family of tumors (EFT) 2001 protocol followed by granulocyte colony-stimulating factor (G-CSF) to avoid febrile neutropenia. He is on regular follow-up with a relapse-free interval of 2 years and 4 months. None of the patients received adjuvant radiotherapy. One patient was lost to follow-up, while another patient refused adjuvant therapy and died with relapse after 8 months of surgery.

## Discussion

Seemayer and colleagues first reported ES/PNET of the kidney in 1975 (2). Renal localization of PNET is very rare (1). Owing to rarity, a limited number of cases have been reported in the literature until now, and hence the proper analysis of prognosis of renal PNET is not available.

Although PNET tumors are mostly seen in adolescents and young adults, these can present in any age group. In our experience, the median age is 43 years, which is higher compared to other studies. Contrary to the literature, men-to-women ratio in our experience was the lowest one. The presenting symptoms of renal PNET are nonspecific, including flank pain, abdominal mass, hematuria, and other symptoms related to genitourinary infections (9). A retrospective study done by Sun et al. showed most patients (87%) having renal PNET on the left side, but our study and case series conducted by Narayanan et al. revealed it to be on the right kidney in most of the patients (10,11).

The imaging characteristics of renal PNET are generally nonspecific. They can masquerade as any other tumor of renal origins such as RCC, Wilms tumor, neuroblastoma, lymphoma, and desmoplastic small RCT.

In 2014, Liu et al. reported the world’s first renal pelvis ES/PNET (12). To our knowledge, very few cases of the renal pelvis, ES/PNET tumor are reported in the literature to date. No known specific imaging findings are available for renal pelvis ES/PNET mimicking as renal pelvis TCC. In our study, one case presented as the tumor of the renal pelvis along with gross hydronephrosis (Figure 1d), which was subsequently confirmed as ES/PNET (Figures 3a, b, and c). The differential diagnosis of renal pelvis ES/PNET includes urothelial cancer, amyloidosis, fibroepithelial polyp, other soft-tissue sarcomas, and lymphoma (13). Preoperative diagnosis is difficult based on imaging. Hence, computed tomography (CT)-guided fine-needle aspiration biopsy and ureteroscopic biopsy are considered for early diagnosis.

In our series, three out of four cases (75%) had some form of IVC involvement. In two cases, level III and IV IVC thrombus were present, and in another case, dense desmoplastic adhesions with IVC were found along the entire length of the mass. In a retrospective study of eight patients done by Seth et al. (Table 2), 50% of the patients had IVC thrombus (14). They reported that the IVC thrombus in PNET was significantly more friable than IVC thrombus of other renal malignancies. Friable IVC tumor thrombus might increase the risk of pulmonary thromboembolism. Renal tumors with IVC thrombus are inherently known to be aggressive biologically. Renal PNET should be suspected in young patients presenting with renal mass and IVC thrombus, and multimodality treatment should be offered, where proven.

**Table 2:** Review of literature for clinical presentation, and treatment and follow-up of renal PNET.

Parameters	Seth et al. 8 cases	Thyavihally et al. 16 cases	Narayanan et al. 7 cases	Sun et al. 8 cases	Our study 4 cases
Age (years)	27.5	27	32	34	43
Sex	M:F: 1:1	M:F: 1.6:1	M:F: 0.75:1	M:F: 1.6:1	M:F: 1:3
Clinical presentation	P-8 (100%) M-8 (100%) H-4 (50%)	P-11 (68.7%) M-6 (37.5%) H-5 (31%)	P-4 (57%) M-3 (42.8%) Incidental finding-1 (14.25%)	Mass-8 (100%) Pain-5 (62.5%) Lower limb oedema-1 (12.5%)	P-03 (75%) M-02 (50%) W-02 (50%) H-01 (25%) F-01 (25%)
Laterality			Right-4 (57%) Left-1 (14.2%) 2 not known	Right-1 (12.5%) Left-7 (87%)	Right-3 (75%) Left-1 (25%)
Extent	L-3 (37.5%) LA-5 (62.5%)	L-10 (63%) LA-01 (6%) Metastasis-05 (31%)	L-03 (42.8%) Metastasis-04 (57%)	L-4 (50%) LA-1 (12.5%) Metastasis-3 (37.5%)	L-2 (50%) L-2 (50%)
IVC involvement	4 (50%)	1 (6.25%)		1 (12.5%)	3 (75%)
Surgery	RN-8 (100%) LND-2 (25%) IVCT-4 (50%)	RN-13 (81%) LND-2 (12.5%) IVCT-1 (6.25%)	RN-6 (85.7%) Renal biopsy-1 (14.2%)	RN-7 (87.5%) LND-7 (87.5%) Needle biopsy-1 (12.5%)	RN-04 (100%) LND-4 (100%) IVCT-02 (50%) IVC cuff excision and repair (25%)
IHC	CD-99 (100%)	CD-99 (100%)	CD-99 (100%)	CD-99: 6 (75%) Vimentin-6 (75%) NSE-6 (75%) WT1-2 (25%) Desmin-1 (12.5%)	CD-99: 04 (100%) FLI-1 03 (75%) Vimentin- 01 (25%)
Adjuvant therapy	CT-6 (75%) RT-2 (25%)	CT-100% RT-9 (56.2%)	CT-5 (71%) RT-4 (57%)	CT-5 (62.5%) (1 case-CIK cell immunotherapy) No adjuvant therapy-3 (37.5%)	CT-01 (25%) RT No
Follow-up	Median follow-up 45 months. Median survival 45 months 3-year DFS 66%	Median follow-up 31 months Median survival 40 months	Follow-up from 6 to 18 months	Median follow-up 16 months Median survival 20 months 3-year DFS 25%	Follow-up up to 2 years and 4 months
Death in the perioperative period or other reasons	02		01 (sepsis at 15 months)		01
Remission	03 (37.5%)	04 (25%)	01 (14.2%)		01 (25%) at 2 years and 4 months
Relapse	03 (42.8%)	07 (43.7%)	04 (57.1%)	08 (100%)	01 (25%) at 8 months
Lost to follow-up			01 (14.2%)		01 (25%)

IVC, inferior vena cava; IHC, immunohistochemistry; PNET, primitive neuroectodermal tumor; LA, locally advanced; RN, radical nephrectomy; LND, Lymph node dissection; IVCT, IVC thrombectomy; NSE, neuron-specific enolase; CIK, cytokine-induced killer; DFS, disease-free survival; CD-99, cluster of differentiation-99.

Preoperative angioembolization may facilitate resection of large and more advanced stage tumors with comparably less blood loss to nephrectomy alone. We will consider it in selected future cases where suspicion of extensive neovascularization is present.

Owing to nonspecific clinical and imaging features, renal PNET diagnosis is mainly based on histopathology and IHC, supported by the cytogenetic study. Renal PNET is characterized by small uniform round cells with dark nuclei, ill-defined cytoplasmic borders, and poorly formed rosette-like structures. Presence of *macrophage inhibitory cytokine* (*MIC-2*) gene products, also known as *CD99, 12E7, E2, 013*, and *HBA71*, suggest a PNET diagnosis (15) to renal PNET. *CD99* is expressed strongly in almost all cases, although it is not specific for renal PNET (16). Besides, nearly two-thirds reveal FLI-1 expression (17). Although FLI-1 protein expression is seen not only in ES/PNET among small blue RCTs, it still can play a valuable adjunctive role in the diagnosis (18). Other markers, such as vimentin, cytokeratin, neuron-specific enolase, and S-100, have also been detected but they are not pathognomonic (19). On IHC, we consistently found CD-99 in all cases and FLI-1 positivity in three (75%) of them.

As per cytogenetic studies, both PNET and Ewing’s sarcoma are associated with translocation of the long arm of chromosomes 11 and 22, t [11, 22] [q22, q12] (19). Both are considered the end of a histological spectrum of “Ewing’s family of tumors” (EFT). The presence of EWSEwing’s sarcoma (EWS) /FLI-1 fusion protein has a decisive and supportive role in diagnosing PNET of the kidney.

The initial extent of disease at presentation is the essential factor while considering treatment. Radical nephrectomy is vital for local control of the disease. However, ES/PNET being an aggressive tumor often relapses even after complete resection. Adjuvant therapy in the form of chemotherapy and radiotherapy increases the chances of survival. The overall 5-year survival was less than 10% before the routine use of chemotherapy for Ewing’s family of tumors. Now survival of 45–55% is reported with multimodality treatment. PNET is treated with similar chemotherapy as Ewing’s sarcoma, as both belong to Ewing’s family of tumors. Earlier, the RCT II protocol was used for PNET, which included 2 mg/m^2^ of vincristine, 75-mg/m^2^ bolus infusion of adriamycin, and 1200 mg/m^2^ of cyclophosphamide (20). With the addition of etoposide and ifosfamide to the VAC regimen, the current standard chemotherapy protocol for PNET has been that for Ewing’s family of tumors. In the EFT-2001 protocol, 1800 mg/m^2^ of ifosfamide per day for 5 days and 100 mg/m^2^ of etoposide per day for the same period are added to the VAC regimen (20).

Sun et al. studied the role of cytokine-induced killer (CIK) cell immunotherapy in a patient of renal PNET (10). They noted a survival advantage of 20 months with lesser adverse events with this form of therapy in comparison to chemotherapy. However, further research is needed to assess the usefulness of CIK cell immunotherapy and other targeted therapies for renal PNET.

In the study done by Thyavihally et al., 11 patients with surgical disease-free status had completed adjuvant treatment. Four out of these were disease-free at the last follow-up period, and a 5-year disease-free survival rate was 36%. In a retrospective study conducted by Sun et al., four cases received adjuvant chemotherapy. They had a significantly better overall survival of 36 months, compared with an overall survival of 10 months in the three patients without adjuvant chemotherapy (10). In the present study, one of the patients with the localized disease received chemotherapy, that is, VAC/IE of a total of 17 cycles and following up regularly with remission for the last 2 years and 4 months. However, another patient with locally advanced disease who refused for adjuvant treatment had a poor outcome with an 8 months survival post-surgery. Thus, adjuvant therapy seems to play a vital role in the management of these cases and has a definitive survival advantage.

Even though the role of radiotherapy is not established in renal PNET, it may be useful in the presence of Gerota’s fascia involvement, positive surgical margins, and locally advanced disease. Kushner et al. from Memorial Sloan Kettering Cancer Center presented a series of 54 cases of extracranial PNETs, which revealed a disease-free survival of 24% at 2 years (21). The radiation dose in their study varied from 5040 to 6000 rads. The uniform radiation therapy approach of Miser et al. has produced reasonable local control in PNET (22). They suggested that radiation therapy might be useful to ablate residual microscopic disease.

Renal PNET is presumed to be more aggressive than the PNET tumor of other locations in the body. It often recurs locally and metastasizes early to the regional lymph nodes, lungs, liver, bone, and bone marrow, resulting in a poor prognosis. Thyavihally et al. in their retrospective study demonstrated median survival to be 40 months, with 3 years and 5 years disease-free survival rate of 60% and 42%, respectively (20). The overall survival in patients who had localized disease was 60 months, compared to patients with infection at regional nodes or distant sites, in whom the survival was 15 months. This study indicates a better chance of survival in localized cases. In a review done by Cuesta Alcalá et al., the mean survival was 10 months. Only three of the 26 subjects had a more prolonged survival: 60, 48, and 24 months (23). The 5-year disease-free survival rate for patients presenting with well-confined extra-skeletal PNET is around 45–55%, and cases with advanced disease at presentation have a median relapse-free survival of only 2 years (6).

In our series, the patient with localized disease received chemotherapy, that is, VAC/IE of a total of 17 cycles and following up regularly with remission for the last 2 years and 4 months. Although statistical comparisons cannot be made due to the small number of cases in our study, we still believe that adjuvant therapy, in addition to radical surgery, has a significant role in survival.

This study is limited by a minimal number of cases, along with attrition and its retrospective nature. Nevertheless, we draw the following conclusion not just from our experience but also from the literature.

## Conclusion

Renal PNET is a distinct clinical entity with aggressive behavior. It can even present as a renal pelvis tumor. Clinical features and imaging findings are nonspecific. Hence, the preoperative diagnosis is challenging. Histopathology and IHC are necessary to diagnose renal PNET. It is an extraordinarily rare disease. Therefore, no definitive treatment guidelines exist for the management. Radical nephrectomy, along with chemotherapy used for Ewing’s family of tumors, provides a definite survival advantage. We conclude that multimodality treatment in the form of surgery, chemotherapy, and radiotherapy should be used for better results.
